# Dasatinib-Induced Pleural and Pericardial Effusions

**DOI:** 10.7759/cureus.19024

**Published:** 2021-10-25

**Authors:** Yousef M Hailan, Ahmed Elyas, Mohammad A Abdulla, Mohamed A Yassin

**Affiliations:** 1 Internal Medicine, Hamad Medical Corporation, Doha, QAT; 2 Cardiology and Cardiovascular Surgery, Heart Hospital, Hamad Medical Corporation, Doha, QAT; 3 Internal Medicine, National Center for Cancer Care and Research, Hamad Medical Corporation, Doha, QAT; 4 Internal Medicine/Hematology, National Center for Cancer Care and Research, Hamad Medical Corporation, Doha, QAT

**Keywords:** dasatinib, pericardial effusion, pleural effusion, cml, chronic myeloid leukemia, tyrosine kinase inhibitor

## Abstract

Chronic myeloid leukemia (CML) is a myeloproliferative disease associated with the Philadelphia chromosome and *BCR-ABL1 *fusion gene. Tyrosine kinase inhibitors (TKI) are now the standard therapy for this condition. Among the approved TKIs for CML is dasatinib. We present a case of a 58-year-old Egyptian male who developed bilateral pleural (grade II) as well as pericardial effusions (grade II) secondary to dasatinib 100 mg once-daily dosing. He was managed by interrupting dasatinib and introducing diuretics and steroids. The objective is to raise awareness about this unfavorable effect as it may affect the patient's quality of life and increase rates of treatment withdrawal.

## Introduction

Chronic myeloid leukemia (CML), a myeloproliferative disease, is associated with Philadelphia (Ph) chromosome t(9;22)(q34;q11), resulting in a *BCR-ABL1* fusion gene. The introduction of tyrosine kinase inhibitors (TKI) has dramatically changed the management of CML. There are over 19 TKIs approved by U.S. Food and Drug Administration (FDA) for hematologic and oncologic malignancies; five of them are approved for CML [1]. The approved TKIs for CML are imatinib, nilotinib, dasatinib, bosutinib, and ponatinib [1].

Dasatinib has been associated with many adverse effects, including fluid retention in the form of pleural and less commonly pericardial effusions. Mechanisms behind that are still largely unclear. The management varies according to the severity and could be supportive for mild cases or invasive with fluid drainage in severe presentations.

We report a case of dasatinib-induced bilateral pleural effusions with pericardial effusion.

## Case presentation

A 58-year-old Egyptian male presented in February 2021 to the emergency department complaining of intermittent chest pain, shortness of breath, and mild cough for three days. The pain is non-radiating, left-sided, and associated with shortness of breath for a few months, worse with deep inspiration. The patient denied headache, blurred vision, palpitations, syncope, no recent history of fever or flu-like symptoms, but reported mild dry cough on deep inspiration. No history of contact with sick or COVID-19 patients.

Past medical history is notable for CML in a complete hematologic response (BCR-ABL1 positive with a BCR-ABL1 to ABL1 percentage ratio of 0.01% [IS] in February 2021), peripheral neuropathy, and benign prostatic hyperplasia. His home prescriptions are dasatinib, pregabalin, amitriptyline, and alfuzosin. Other parts of history were non-contributary.

**Figure 1:**
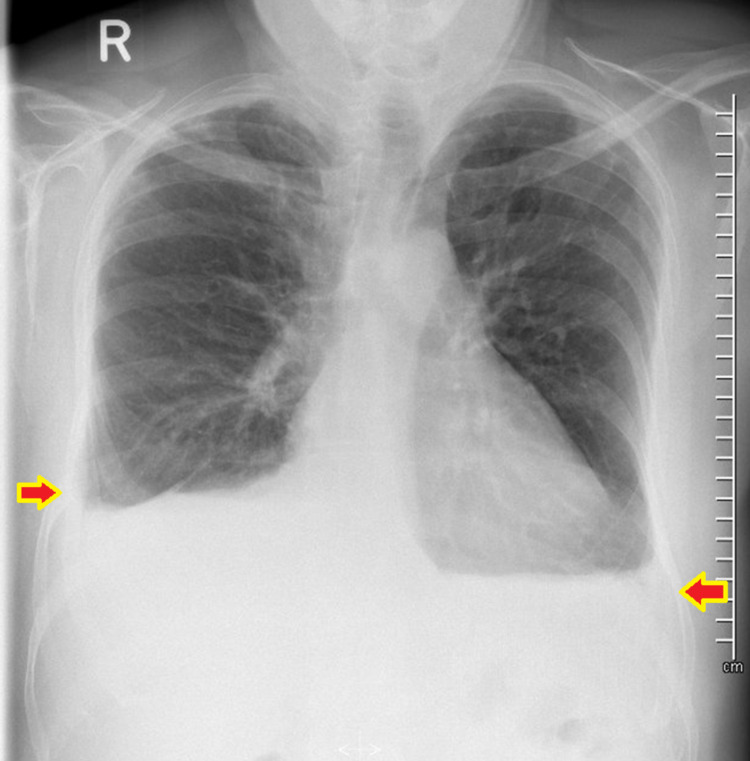
Chest x-ray showing bilateral pleural effusion and blunting of both costophrenic angles (August 2020)

Before this admission, he was followed by hematology and cardiology outpatient departments for shortness of breath. He was found to have grade II bilateral pleural effusion (Figure 1). He was on dasatinib 100 mg daily, and he was advised to reduce the dose to 50 mg and start taking furosemide. Outpatient cardiology workup with electrocardiography (ECG), transthoracic echocardiography (TTE), and Tc 99 MIBI Adenosine myocardial perfusion stress tests was negative except for mild pericardial effusion.

**Figure 2:**
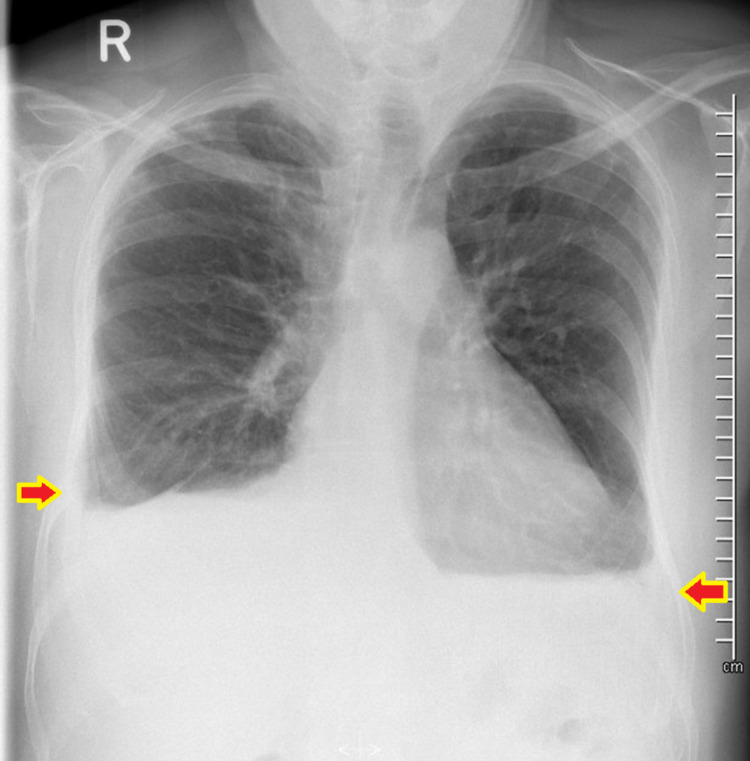
Chest x-ray showing bilateral pleural effusion (February 2021)

**Figure 3:**
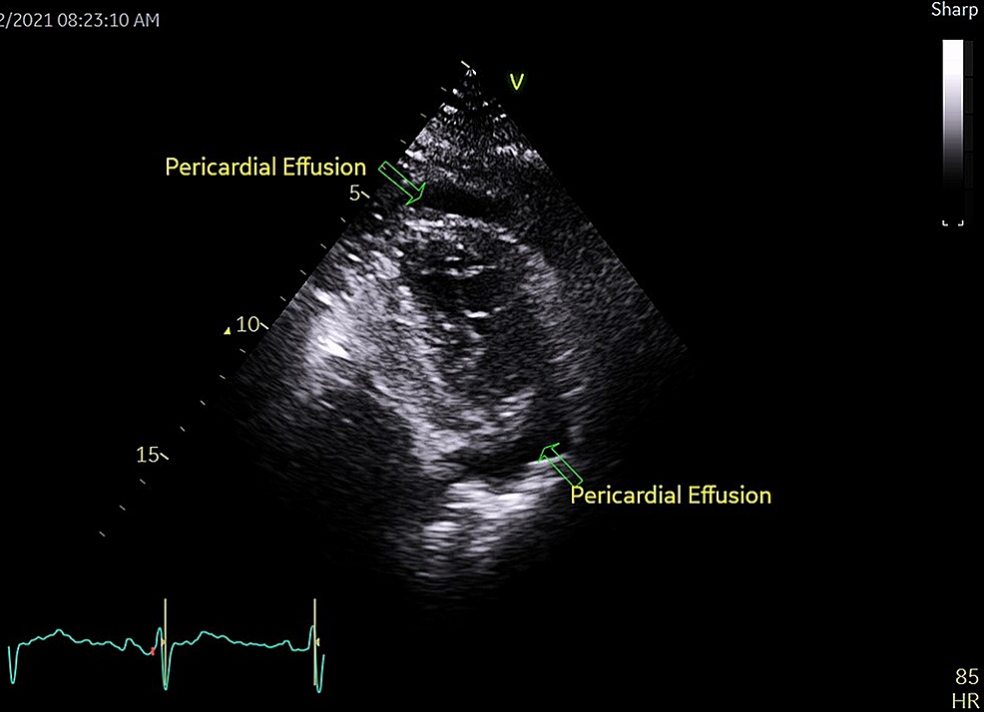
Transthoracic echocardiography images (February 2021)

On this admission, physical examination showed bilaterally reduced breath sounds and dullness to percussion in both lower zones. Blood investigations were within normal ranges, including CBC, electrolytes, troponins, NT pro-BNP, renal and liver function tests, as well as thyroid function tests. Chest x-ray showed bilateral pleural effusion (Figure 2). CT pulmonary angiogram (CTPA) ruled out pulmonary embolism and revealed marked right and moderate left-sided pleural effusions and significant pericardial effusion. Further evaluation with TTE confirmed circumferential mild to moderate (grade II) pericardial effusion (Figure 3). SARS-CoV-2 RT-PCR was negative as well.

 

Findings of diagnostic pleurocentesis were consistent with lymphocytic exudative picture (pleural fluid WBC 1,750/µL, lymphocyte 86.0%, protein 46.0 g/L, albumin 31.4 g/L, while total serum protein is 78 g/L, and LDH is 293 U/L). However, pleural fluid cultures and TB workup, including TB culture, were negative. The patient was treated with ceftriaxone (2 g IV q24hrs) and azithromycin 500 mg (q24 hrs) for suspected chest infection. During the hospital course, the patient remained afebrile.

**Figure 4:**
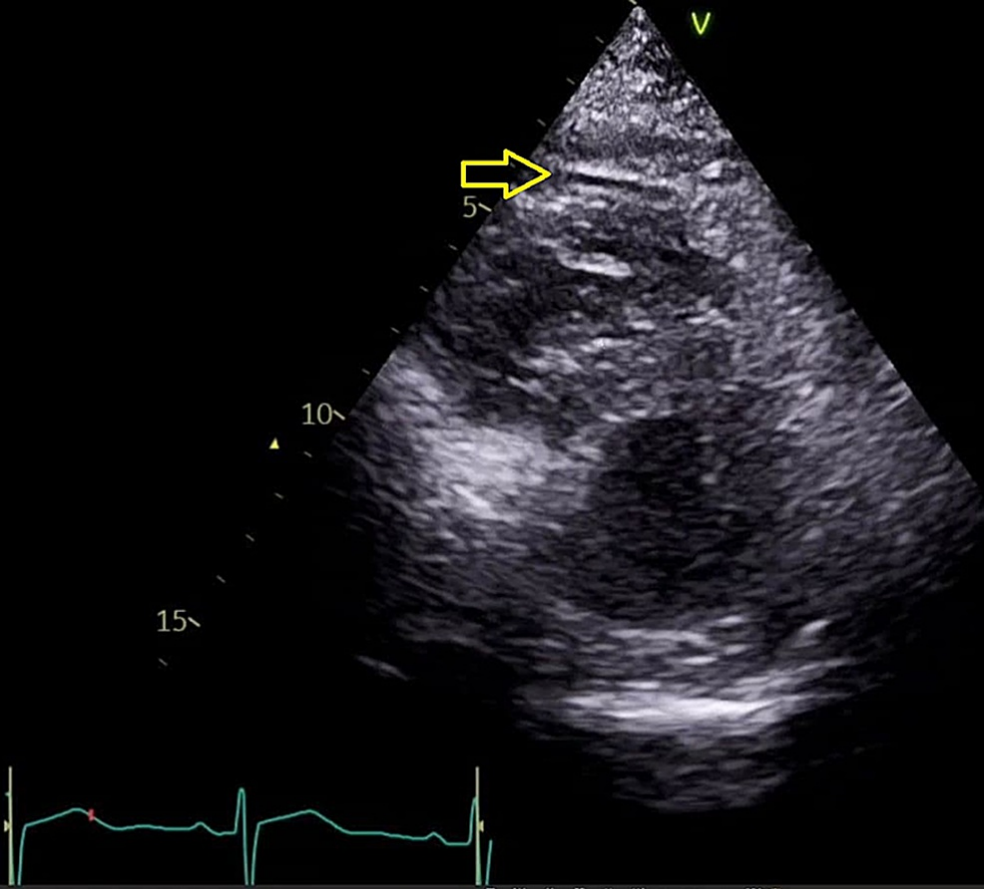
Follow-up transthoracic echocardiography (parasternal short axis view) showing minimal pericardial effusion denoting improvement (April 2021)

At this point, the symptoms resolved, and the effusions were attributed to dasatinib. Thus, it was held, and the plan was a trial of a small dose of diuretics (furosemide) with a short course of steroids (prednisolone 40 mg for five days - extended for another week later). The response followed via repeating chest x-ray and echocardiography as an outpatient. Repeated echocardiogram showed minimal pericardial effusion, and chest x-ray showed minimal pleural effusion, denoting improvement (Figure 4). The patient was shifted to imatinib 400 mg once daily.

## Discussion

Dasatinib, a second-generation TKI, proved effective in achieving major or complete cytogenic responses in chronic phase CML [2]. It shows superior and more profound responses in comparison with imatinib. Although dasatinib is generally well tolerated, several hematologic and non-hematologic side effects have been reported. Among the reported adverse effects is the development of pleural effusions in up to 14%-35% [3,4]. The likely mechanism - through which dasatinib causes pleural effusion - is still unclear. However, it may be related to inhibition of the platelet-derived growth factor receptor beta (PDGFRβ), which plays a part in the regulation of angiogenesis [3,5,6]. Another proposed mechanism is autoimmune-mediated [4,7]. Treatment of dasatinib-induced pleural effusions is dose interruption, introducing diuretics with or without steroids [8-10]. Later on, dasatinib may be resumed, depending on the severity of the initial presentation, at a lower dose [8,10].

On the other hand, pericardial effusion is less reported [11-14], and mechanisms and management are less established. In a report of 23 patients who received dasatinib, nine patients developed grade I/II pleural effusions, and only one case developed pericardial effusion (grade I/II) [11]. Another center reported that out of 13 patients on dasatinib, nine patients developed fluid retention, two of them had pericardial effusion [12]. The latter two patients were taking dasatinib 100 mg/day. We assume it shares common pathogenesis with pleural effusions. For the most part, it is managed in a similar way to pleural effusions.

The risk for developing the two complications mentioned above is thought to increase in older patients, those who have multiple comorbidities, especially cardiac or renal, autoimmune diseases, advanced CML, or taking higher doses of dasatinib [8,9]. A review of 670 patients comparing dasatinib dosing of 100 mg once daily to 70 mg twice daily concluded that a dose of 100 mg once daily is associated with similar efficacy, better tolerability, and fewer adverse events, especially lower rates of pleural effusions [15]. A smaller study, on nine patients with chronic phase CML with resistance or intolerance to imatinib, compared the efficacy and safety of 50 mg once daily dose of dasatinib with the higher 100 mg daily dose settled that it may be safe and effective with a better safety profile [16].

Our patient was diagnosed with CML in January 2018 and was on dasatinib as front-line therapy (Sokal score 0.8 - Intermediate risk). He was taking 100 mg once daily dosing of dasatinib. His first presentation to the clinic with the above complaint was in August 2020. He was counseled to reduce the dose to 50 mg once daily and to be started on diuretics. The patient did not comply with this advice, and his symptoms progressed. On further retrospective review of his charts over the previous two years, it was found that his chest x-ray and echocardiography have shown evidence of pericardial and pleural effusion after starting dasatinib therapy which had worsened gradually over time. His first echocardiography was positive for a mild pericardial effusion (grade I) since May 2018, and his chest x-ray showed evidence of cardiomegaly on April 2020 (denoting pericardial effusion) with no evident pleural effusion.

We adopted the National Cancer Institute (NCI) Common Terminology Criteria for Adverse Events (AE) version 5.0 grading scale in our patients. A grade II severity in pleural effusion is defined as symptomatic effusion needing intervention such as diuretics or therapeutic thoracocenteses [17]. In comparison, grade II on the scale for pericardial effusion is small to moderate size asymptomatic effusion without physiologic consequences or needing emergency interventions [17].

Ruling out mycobacterial tuberculosis (TB) infection was necessary for several reasons. First, the high prevalence of TB among Middle Eastern and North African patients. The second, the lymphocytic exudative picture of the pleural effusion in our patient. Third, compared to the general population, TB occurs up to 9 times more in patients with hematologic malignancies [18]. Lastly, the treatment implications and possible drug interactions with anti-TB medications.

In our patient, we believe that chest x-rays have shown early evidence of pericardial effusion shown by cardiomegaly with a globular heart which can be followed in those cases to detect evidence of pericardial effusion as well as pleural effusion. Also, the lymphocytic exudative results of the diagnostic pleural tapping are consistent with other case reports [19,20]. In managing our patient, we held dasatinib and began diuretic therapy. A component of autoimmune pathogenesis can be suspected in our patient in the view of his hypothyroidism. Thus, steroids were also given.

## Conclusions

 Physicians should be alert to this complication in patients on dasatinib therapy as it may affect the patient's quality of life and increase rates of treatment withdrawal. Also, dasatinib 100 mg once daily dosing may not be a proper dose for everyone, and more studies are needed to evaluate the efficacy and safety profile of smaller doses. It is also important to assess patients' comorbidities to identify higher-risk patients for such complications. In addition to physical examination, serial chest x-ray can be a helpful tool for patients on dasatinib therapy to detect pericardial and pleural effusion as having easier access and higher availability compared to echocardiography.
